# RNA sequencing-based analysis of gallbladder cancer reveals the importance of the liver X receptor and lipid metabolism in gallbladder cancer

**DOI:** 10.18632/oncotarget.9181

**Published:** 2016-05-05

**Authors:** Mingxin Zuo, Asif Rashid, Ying Wang, Apurva Jain, Donghui Li, Anu Behari, Vinay Kumar Kapoor, Eugene J. Koay, Ping Chang, Jean Nicholas Vauthey, Yanan Li, Jaime A. Espinoza, Juan Carlos Roa, Milind Javle

**Affiliations:** ^1^ Department of Gastrointestinal Medical Oncology, University of Texas MD Anderson Cancer Center, Houston, Texas, USA; ^2^ Department of Pathology, University of Texas MD Anderson Cancer Center, Houston, Texas, USA; ^3^ Department of Bioinformatics and Computational Biology, University of Texas MD Anderson Cancer Center, Houston, Texas, USA; ^4^ Department of Surgical Gastroenterology, Sanjay Gandhi Post-Graduate Institute of Medical Sciences (SGPGIMS), Lucknow, UP, India; ^5^ Department of Radiation Oncology, University of Texas MD Anderson Cancer Center, Houston, Texas, USA; ^6^ Department of Surgical Oncology, University of Texas MD Anderson Cancer Center, Houston, Texas, USA; ^7^ SciLifeLab, Division of Translational Medicine and Chemical Biology, Department of Medical Biochemistry and Biophysics, Karolinska Institutet, Solna, Stockholm, Sweden; ^8^ Department of Pathology, Advanced Center for Chronic Diseases (ACCDiS), UC-Center for Investigational Oncology (CITO), School of Medicine, Pontificia Universidad Católica de Chile, Santiago, Chile

**Keywords:** RNA sequence, gallbladder cancer, liver X receptor, lipid metabolism pathways

## Abstract

Gallbladder cancer (GBC) is an aggressive malignancy. Although surgical resection may be curable, most patients are diagnosed at an advanced unresectable disease stage. Cholelithiasis is the major risk factor; however the pathogenesis of the disease, from gallstone cholecystitis to cancer, is still not understood. To understand the molecular genetic underpinnings of this cancer and explore novel therapeutic targets for GBC, we examined the key genes and pathways involved in GBC using RNA sequencing. We performed gene expression analysis of 32 cases of surgically-resected GBC along with normal gallbladder tissue controls. We observed that 519 genes were differentially expressed between GBC and normal GB mucosal controls. The liver X receptor (LXR)/retinoid X receptor (RXR) and farnesoid X receptor (FXR) /RXR pathways were the top canonical pathways involved in GBC. Key genes in these pathways, including *SERPINB3* and *KLK1*, were overexpressed in GBC, especially in female GBC patients. Additionally, *ApoA1* gene expression suppressed in GBC as compared with normal control tissues. LXR and FXR genes, known to be important in lipid metabolism also function as tumor suppressors and their down regulation appears to be critical for GBC pathogenesis. LXR agonists may have therapeutic value and as potential therapeutic targets.

## INTRODUCTION

GBC is an aggressive but uncommon malignancy affecting about 5000 individuals in the United States annually. Although the incidence of this disease is decreasing in the Western Hemisphere, it continues to pose a challenge in certain geographic locations such as in Latin America and Asia. [1, 2] Gallstone is a known risk factor for this cancer. However, the actual incidence of GBC in patients with gallstones is very low and 10-15% of adults in the western world have gallstones. Other risk factors include *Salmonella typhi* infection, primary sclerosing cholangitis, obesity, and gallbladder polyps. Still unknown is why certain ethnic groups have a higher predisposition to GBC than others. Molecular characterization of this cancer has been very limited thus far. We investigated the genetic variants in GBCs and compared them with in normal gallbladder tissue using the powerful RNA sequencing (RNA-seq) technology to better understand the pathogenesis of this disease and define targets for its therapy.

## RESULTS

### RNA-seq: gene expression alterations in GBC cases

**Figure 1:**
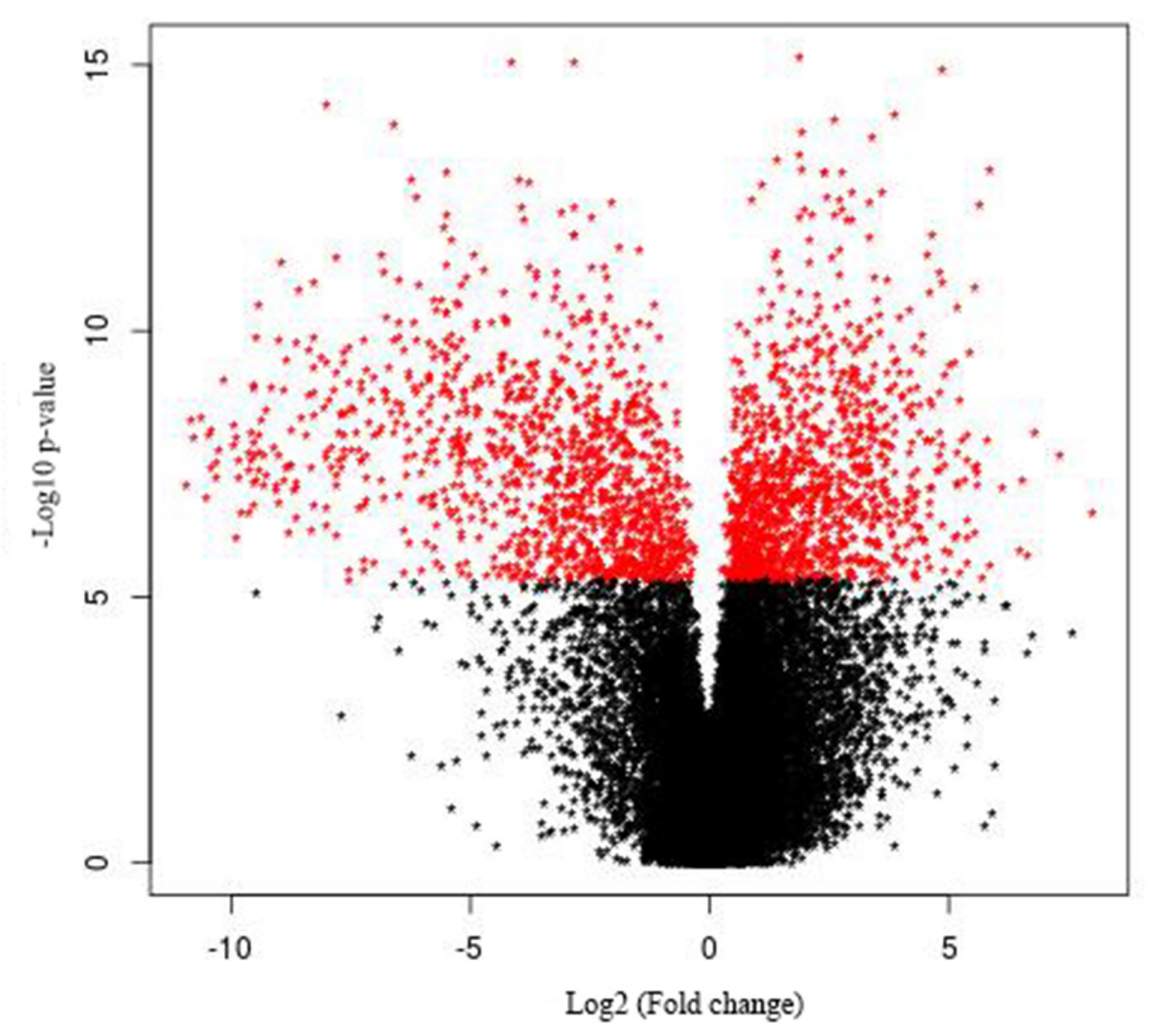
Volcano plot of gallbladder cancer vs. control The 519 differentially expressed genes that fall above our threshold value are pictured in red.

**Figure 2:**
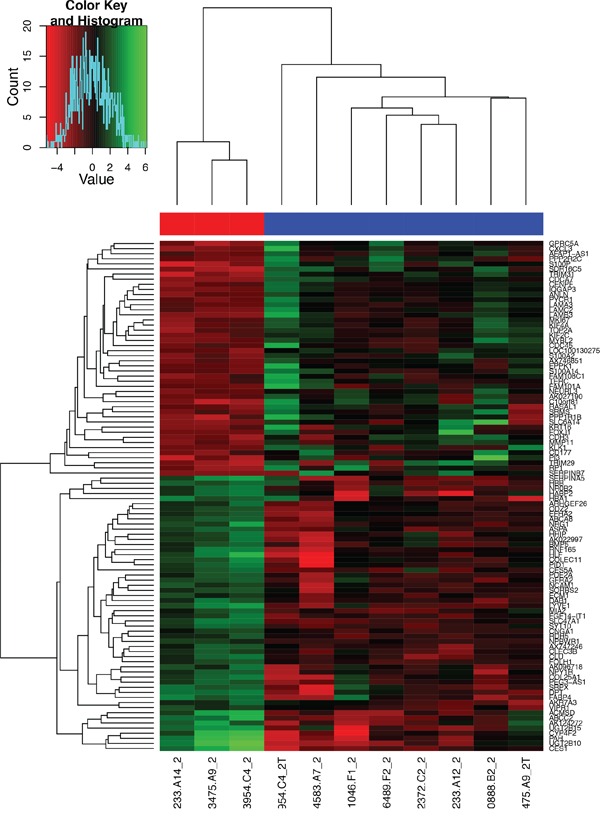
Heat map of the 100 most overexpressed (red) and underexpressed (green) genes in the formalin-fixed, paraffin-embedded GBC specimens The depth of the colors reflects the degree of upregulation and downregulation: the deeper the color, the more extreme the log fold-change in expression.

We analyzed alterations in gene expression in eight GBC and three normal gallbladder tissue specimens using RNA-seq. We identified 519 genes as being differentially expressed in the GBC samples as compared with normal GB tissue (*p* < 0.05) (Figure 1, Supplemental Table 1). The heat map (Figure 2) depicts the 100 most frequently overexpressed and underexpressed genes in the GBC specimens. These genes were ranked according to log fold-change. Table 1 lists the top upregulated and down-regulated genes in the GBC specimens tested.

**Table 1:** Fold-change in gene expression in GBC specimens

Gene	Description	Fold-change (log ratio)
SERPINB3	Serpin peptidase inhibitor, clade B (ovalbumin), member 3	9.981
DUSP1	Dual-specificity phosphatase 1	9.504
FOXJ1	Forkhead box J1	9.236
CHP1	Calcineurin-like EF-hand protein 1	9.124
KLK5	Kallikrein-related peptidase 5	8.476
HOXB13	Homeobox B13	8.414
MAGEB2	Melanoma antigen family B, 2	8.211
KLK1	Kallikrein-related peptidase 1	7.974
HOXC10	Homeobox C10	7.816
CLCA4	Chloride channel accessory 4	7.767
DCAF12L1	DDB1- and CUL4-associated factor 12-like 1	−8.218
PPAN-P2RY11	PPAN-P2RY11 readthrough	−8.041
MT1A	Metallothionein 1A	−7.726
ZNF275	Zinc finger protein 275	−7.636
GIMAP1-5	GIMAP1-GIMAP5 readthrough	−7.572
MT1M	Metallothionein 1M	−7.342
APOA1	Apolipoprotein A-I	−7.292
PGLYRP2	Peptidoglycan recognition protein 2	−7.274
SHBG	Sex hormone-binding globulin	−7.120
CYP1A1	Cytochrome P450, family 1, subfamily A, polypeptide 1	−6.770

### Ingenuity pathway analysis (IPA)

**Figure 3:**
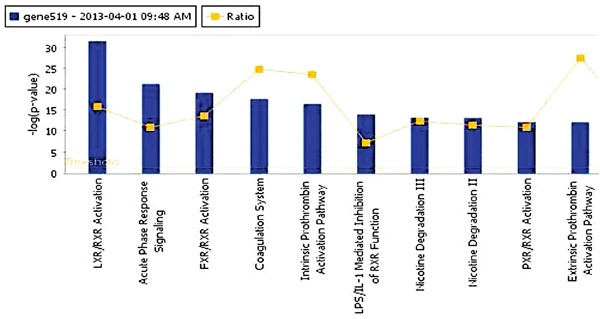
The top 10 canonical pathways that differed in GBC and normal gallbladder tissue specimens

**Figure 4:**
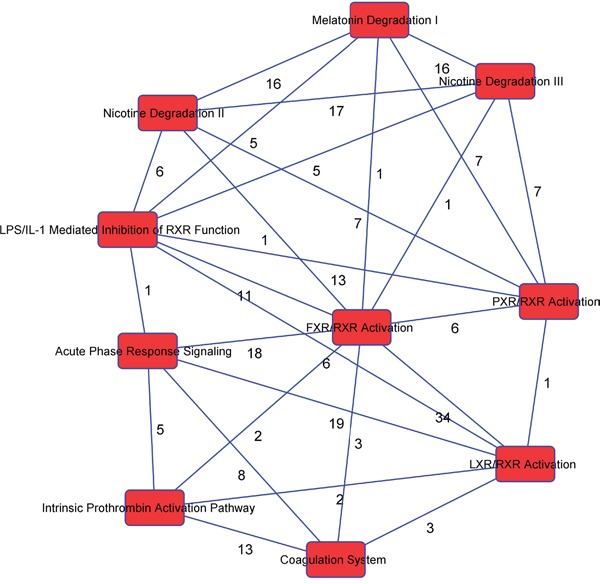
The interaction network analysis of key pathways in GBC

We used IPA to identify the significantly different pathways in GBC and normal gallbladder tissue specimens and used log fold-change as a major observation of the cell signaling pathways analysis. Of the 519 significantly different genes, 503 genes were matched in the IPA database. Figure 3 shows the top 10 canonical pathways based on the 500 significant genes that differed in the GBC and normal gallbladder specimens. The IPA results demonstrated that the alteration of the LXR/RXR and FXR/RXR pathways occurring as a result of LXR and FXR down regulation play important roles in GBC (Table 2). Also, the results show the lipid metabolism pathway and gallbladder cell transport system are critical in gallbladder cancer pathogenesis. The causal network relationship of the top canonical pathways is shown in Figure 4.

**Table 2:** Top canonical signaling pathways in gallbladder cancer

Pathway	*p*	Ratio
LXR/RXR activation	3.73 E-32	39/136 (0.287)
Acute-phase response signaling	6.58 E-22	35/179 (0.196)
FXR/RXR activation	9.14 E-20	25/101 (0.248)
Coagulation system	2.58 E-18	17/38 (0.447)
Intrinsic prothrombin activation	3.79 E-17	15/35 (0.429)

### Ingenuity pathway analysis (IPA)

We used IPA to identify the significantly different pathways in GBC and normal gallbladder tissue specimens and used log fold-change as a major observation of the cell signaling pathways analysis. Of the 519 significantly different genes, 503 genes were matched in the IPA database. Figure 3 shows the top 10 canonical pathways based on the 500 significant genes that differed in the GBC and normal gallbladder specimens. The IPA results demonstrated that the alteration of the LXR/RXR and FXR/RXR pathways occurring as a result of LXR and FXR down regulation play important roles in GBC (Table 2). Also, the results show the lipid metabolism pathway and gallbladder cell transport system are critical in gallbladder cancer pathogenesis. The causal network relationship of the top canonical pathways is shown in Figure 4.

### Validation of next-generation sequencing data using RT-PCR

**Figure 5:**
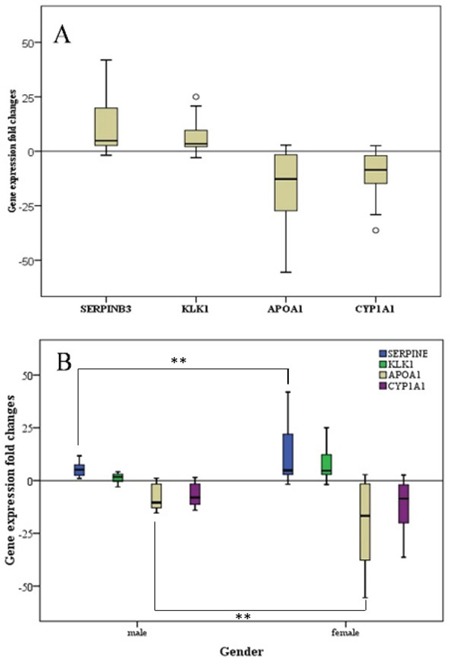
Fold-change in gene expression in GCB patients **A.** The fold-change in all patients (*n* = 32). **B.** The fold-change in female patients (*n* = 21) compared with that in male patients (*n* = 11). **p < 0.01.

To validate the next-generation sequencing findings, we extracted total RNA from the 32 formalin-fixed, paraffin-embedded (FFPE) GBC and normal gallbladder tissue specimens. We assayed five upregulated genes and four down-regulated genes with different biologic functions and involvement in diverse molecular pathways. These genes consisted of overexpressed (*SERPINB3, DUSP1, CHP1, KLK5, and KLK1*) and underexpressed (*MT1A, MT1M, APOA1*, and *SHBG*) genes. RT-PCR data on the expression of these genes were normalized according to those on the expression of glyceraldehyde-3-phosphate dehydrogenase (GAPDH). The results demonstrated that *SERPINB3* and *KLK1* were expressed at higher levels and *APOA1* was expressed at lower levels in GBC than in normal gallbladder tissue specimens. Furthermore, *SERPINB3* expression was markedly higher in female than in male patients (Figure 5).

## DISCUSSION

Our study suggests that unique genetic events result in alteration of the LXR/RXR and FXR/RXR pathways in GBC cells. LXRs are nuclear receptor family members that function in cholesterol transport, glucose metabolism and the modulation of inflammatory responses. There is now strong evidence to support the involvement of LXR and FXR in a variety of malignancies as tumor suppressors and the potential efficacy of their ligands in these diseases. FXR is a bile acid-activated transcription factor that is involved in metabolic regulation in the gut-liver axis. LXR and FXR play critical roles in bile acid homeostasis, glucose regulation and lipid metabolism.

Cancer cells depend on increased cholesterol biosynthesis for active signaling and tumor growth. The sterol response element-binding proteins are the master transcriptional regulators of cholesterol and fatty acid pathway. Nuclear sterol receptors (LXR and FXR) coordinate with these molecules when cholesterol is available. LXR and FXR function as intracellular sensors for sterols and bile acids, respectively. In addition, LXR/RXR is a “permissive heterodimer” that may be activated by either an LXR agonist or RXR ligand. The LXR/RXR heterodimer binds to a direct repeat 4 response element in regulatory regions of their target genes, such as *ABCA1*, *ABCG5*, and *APOE* [3, 4]. LXRs may also bind to all three peroxisome proliferator-activated receptor (PPAR) subtypes (PPARα, PPARγ, and PPARδ) with different binding affinities. PPARs, which are activated by free fatty acid, are widely accepted as having roles in metabolic pathways and inflammation. Mounting evidence demonstrates that PPARs repress nuclear factor NF-κB, signal transducer and activator of transcription, and AP-1 target genes in response to a variety of inflammatory stimuli. [5–7]

Prior studies have described the association of these pathways in the development of gallstones, the primary risk factor associated with GBC. LXR-β polymorphisms influence GBC susceptibility through estrogen and gallstone-dependent pathways [8]. LDLR-mediated hepatic cholesterol uptake and intestinal cholesterol absorption play important roles in LXR-promoted lithogenesis. [9] The ability of these receptors linking metabolism with inflammatory signaling makes them potentially attractive targets for treatment of metabolic diseases, inflammation, and cancer.

Many recent studies report the antiproliferative effect of LXR activation in colon [10–13], prostate [14–16], breast [17, 18], and pancreatic [19] cancer and GBC [20] cells. Deletion of both LXRβ alleles in female mice leads to a wide range of preneoplastic lesions in the gallbladder, such as dysplasia, metaplasia, hyperplasia, and adenomas on a background of chronic cholecystitis. [20] Interestingly, ovariectomy prevented the formation of these lesions, providing experimental evidence for an hormonal regulation of GBC progression and the well-known epidemiological association that women have a higher risk of developing GBC than men [21]. Importantly, these appeared in the absence of gallstones, suggesting a potential role for LXRβ in gallbladder carcinogenesis irrespective of lithiasis. [20] The inhibitory effects of LXR activation on cancer cells may be related to alteration of tumor metabolism, microenvironment, key growth pathways, and activation of apoptotic processes. [22] FXR also appears to be critical in the development of liver tumors. Huang et all showed that FXR ^−/−^ mice spontaneously developed liver tumors. [23] Given the critical roles of these receptors in various diseases, researchers have identified many natural and synthetic agonists of the LXRs. These agonists include the synthetic LXR agonist T0901317 [24–27] and the more LXR-selective agonist GW3965 [28], the partial and tissue-selective LXRβ-specific agonist WYE-672, and the intestine-specific ligand GW6340 without lipogenic activities. [7, 29, 30] Investigators have extensively studied the first-generation LXR agonists (T0901317 and GW3965) in various cancers, including colon, prostate, pancreatic, breast, and lung cancer. [31]

Our results demonstrated that the *SERPINB3* and *KLK1* genes were highly expressed in GBC specimens. SERPINB3 is a member of the ovalbumin-serine protease inhibitor family whose expression is upregulated in many advanced cancers with poor prognoses, including breast, lung, ovarian, and liver cancers [32–35]. Researchers detected high levels of *SERPINB3* expression in primary liver tumors but not in normal liver tissue and showed a significant correlation with transforming growth factor-β1 and cytoplasmic β-catenin expression in hepatocellular carcinomas with poor prognoses. Furthermore, high levels of *SERPINB3* expression have been significantly associated with early tumor recurrence [36]. SERPINB3 also induces epithelial-mesenchymal transition and cell proliferation associated with downregulation of E-cadherin expression and increased β-catenin expression. High expression of SERPIN3, therefore, maybe a marker of prognostic relevance in GBC. *KLK1* is a member of the human kallikrein family and is functionally conserved in its capacity to release the vasoactive peptide and Lys-bradykinin. The kallikreins are serine proteases that have been recognized as cancer biomarkers and have also been implicated in cancer-related processes, including cell-growth regulation, angiogenesis, invasion and metastasis.

Our study has several limitations. While we hypothesize the role of LXR and FXR dimers in GBC pathogenesis, the nature of the pathogenic role of LXR suppression is not yet demonstrated in this observational, non-mechanistic study. Furthermore, our sample size is limited. However, the validation of our findings in a larger cohort adds to the strength of our findings.

In conclusion, this RNA sequencing analysis highlights the role of metabolic alterations in GBC and the potential benefit of targeting LXR/RXR pathway in this disease. LXR suppression may have an important pathogenic roles in GBC. LXR agonist may have clinical and therapeutic implications.

## MATERIALS AND METHODS

### Patients and tissues

The specimens used in this study were obtained from formalin-fixed, paraffin-embedded specimens from 32 patients with gallbladder carcinoma and controls (11 male and 21 female) at MD Anderson Cancer Center and Professor Kapoor's laboratory. RNA-seq was performed for 11 cases, and reverse transcription (RT)-polymerase chain reaction (PCR) analysis was used to confirm these aberrant gene expressions found in RNA-seq in all 32 cases. All GBC specimens were confirmed by a pathologist at The University of Texas MD Anderson Cancer Center. Patient demographics, clinical and survival data, and treatment history were retrieved from the patients' medical records. The study protocol was approved by the MD Anderson Institutional Review Board. Patient characteristics, including sex, age, GBC stage, and histology, are listed in Table 3.

**Table 3:** Patient characteristics

Case number	Age at diagnosis, years	Sex	Ethnicity	GBC stage at diagnosis	Histology
1	49	F	White	IIIA	Adenocarcinoma
2	61	M	Hispanic	IIIA	Adenocarcinoma
3	45	M	Hispanic	IVA	Adenocarcinoma
4	52	F	Black	II	Adenocarcinoma
5	55	F	White	IVB	Adenocarcinoma
6	70	F	Hispanic	II	Adenocarcinoma
7	66	F	Hispanic	II	Adenocarcinoma
8	84	M	White	IIIA	Adenocarcinoma
9	62	F	White	IIIB	Adenocarcinoma
10	62	F	Hispanic	III A	Adenocarcinoma
11	56	F	White	II	Adenocarcinoma
12	53	M	White	IVB	Adenocarcinoma
13	74	F	White	IIIA	Adenocarcinoma
14	77	F	Hispanic	IIIB	Neuroendocrine
15	55	F	Hispanic	II	Adenocarcinoma
16	61	M	White	IIIB	Adenocarcinoma
17	47	M	White	II	Adenocarcinoma
18	44	M	White	IIIB	Adenosquamous
19	58	M	White	IIIB	Adenosquamous
20	59	F	White	IIIB	Adenosquamous
21	68	M	White	II	Adenocarcinoma
22	58	F	White	IIIA	Adenocarcinoma
23	48	F	Black	II	Adenocarcinoma
24	73	F	White	IIIB	Adenocarcinoma
25	71	F	Hispanic	IIIA	Adenocarcinoma
26	68	F	Hispanic	II	Adenocarcinoma
27	82	F	White	II	Adenocarcinoma
28	55	F	White	IIIB	Adenocarcinoma
29	51	F	Black	IIIB	Adenocarcinoma
30	84	F	White	IIIA	Adenocarcinoma
31	58	M	White	II	Adenosquamous
32	61	M	White	IIIB	Adenocarcinoma

### RNA-seq analysis [37–39]

One slide from each specimen had been stained with hematoxylin and eosin and marked by a pathologist to ensure that the tissue section contained more than 80% tumor cells for macrodissection. Two sets of slides containing both normal GB and GBC tissue were scraped and poled into two separate tubes. These pooled specimens were then subjected to RNA isolation. An RNeasy kit (QIAGEN, Valencia, CA) was used for total RNA preparation. RNA samples were converted into cDNA libraries using a TruSeq Stranded Total RNA sample preparation kit (Illumina, San Diego, CA). Briefly, total RNA samples were concentration-normalized, and ribosomal RNA was removed using biotinylated probes that selectively bind ribosomal RNA species. This preserved messenger RNA and other noncoding RNA species, including long noncoding RNA, small nuclear RNA, and small nucleolar RNA. The resulting ribosomal RNA-depleted RNA was fragmented using heat in the presence of divalent cations, with fragmentation times varying according to input RNA degradation. Fragmented RNA was converted into double-stranded cDNA, with dUTP used in place of dTTP in a second-strand master mix. A single base was added to the cDNA, and forked adaptors that included index, or barcode, sequences were attached via ligation. The resulting molecules were amplified via PCR for 15 cycles. During PCR, the polymerase stalled when a dUTP base was encountered in the template. Final libraries were quantified via PCR, normalized to 2 nM, and pooled. Pooled libraries were bound to the surface of a flow cell, and each bound template molecule was clonally amplified up to 1000-fold to create individual clusters. Four fluorescently labeled nucleotides were then flowed over the surface of the flow cell and incorporated into each nucleic acid chain. Each nucleotide label acted as a terminator for polymerization. The fluorescence of each cluster was measured during the base identification. Dye was then enzymatically removed to allow for incorporation of the next nucleotide during the next cycle.

### RNA extraction and quantitative real-time RT-PCR

Total RNA was isolated from the 32 GBC-patient FFPE tissue specimens. RNA was extracted as performed in a next-generation sequencing assay. cDNA was synthesized from 1 μg of total RNA using an iScript cDNA Synthesis Kit (Life Technologies, Hercules, CA). Real-time RT-PCR analysis was performed using a QuantiFast SYBR Green PCR Kit (QIAGEN). The primer sequences were synthesized by Sigma (St. Louis, MO). Gene expression levels were normalized according to the average cycle threshold values for the internal control gene glyceraldehyde-3-phosphate dehydrogenase. Cycle threshold values were extracted using the SDS 2.3 software program (Applied Biosystems, Carlsbad, CA). Data analysis was performed using the ΔΔCt method. The primer sequences used in quantitative RT-PCR are listed in Table 4.

**Table 4:** Primer sequences used in quantitative RT-PCR analysis

Gene		Sequence 5′ to 3′
SERPINB3	Forward	5′-GCA AAT GCT CCA GAA GAA AG-3′
	Reverse	5′-CGA GGC AAA ATG AAA AAG ATG-3′
DUSP1	Forward	5′-CCT GAC AGC GCG GAATCT-3′
	Reverse	5′-GAT TTC CAC CGG GCC AC-3′
CHP1	Forward	5′-CCA GAG GAT TCC AGA ACT TGC C-3′
	Reverse	5′- GAA TCC TCG GAA GTT TAC CTG ATC −3′
KLK5	Forward	5′-CCG GTG ACA AAG CAG GTA GAG −3′
	Reverse	5′-GTG AAC TTG CAG AGG TTG GTG TA −3′
KLK1	Forward	5′-GGA CTA CAG CCA CGA CCT CAT GCT GC-3′
	Reverse	5′-GTC GGG GAA TTC GAA GTC GTC TGG-3′
MT1M	Forward	5′-TTA TTT GGT GTA TAG TTT TTT TTG T-3′
	Reverse	5′-TAA ACC CAA CAT AAA TAC CAA ACA-3′
APOA1	Forward	5′-CCC AGT TGT CAA GGA GCT TT-3′
	Reverse	5′-TGG ATG TGC TCA AAG ACA GC-3′
SHBG	Forward	5′-ACT CAG GCA GAA TTC AAT CTC −3′
	Reverse	5′- CTT TAA TGG GAA GCG TCA GT-3′
CYP1A1	Forward	5′-TCC AAG AGT CCA CCC TTC C-3′
	Reverse	5′-AAG CAT GAT CAG TGT AGG GAT CT-3′

### IPA

Ingenuity Pathway Analysis (IPA; QIAGEN) was used to identify the pathways that differed significantly in the GBC and normal gallbladder tissue specimens. A *p* value cutoff of 0.0001 was used to identify statistically significant difference in networks. This analysis was performed to identify gene interactions within these networks.

### Bioinformatics and statistical analysis

The bioinformatic software program mRNAv7-RSEM (Quintiles, Durham, NC) was used to analyze RNA-seq data. To prepare the reads for alignment, the sequencing adapters and other low-quality bases were clipped. RSEM was used to quantify genes and transcripts The RSEM v1.1.18 program rsem-calculate-expression was run with parameters optimized for Illumina 50 × 50 paired-end sequencing. The University of California, Santa Cruz Known Gene transcriptome was used. Sustained misalignment of reads with the transcriptome may have resulted from missed or a lack of annotation or genomic DNA. To determine the origin of all reads as a method of quality control, the unaligned reads were aligned with the full genome (not the transcriptome) using BWA3. For cross-sample analysis, upper-quartile normalization of the read counts was performed. Two group comparisons were performed using edgeR [40, 41] and moderated separately using *t*-tests. The more conservative *p* value was used for any given gene. Heat maps were created using the R statistical programming language.

## SUPPLEMENTARY TABLE





## Abbreviations

FXR: farnesoid X receptor
GBC: gallbladder cancer
IPA: Ingenuity Pathway Analysis
LXR: liver X receptor
PCR: polymerase chain reaction
PPAR: peroxisome proliferator-activated receptor
RNA-seq: RNA sequencing
RT: reverse transcription
RXR: retinoid X receptor
