# Supplementary material for: RNA sequencing-based analysis of gallbladder cancer reveals the importance of the liver X receptor and lipid metabolism in gallbladder cancer
Source: Oncotarget. 2016 May 5;7(23):35302–12. doi: 10.18632/oncotarget.9181 (PMC5085230; doi:10.18632/oncotarget.9181)
Supplement: Supplementary file 1 [file oncotarget-07-35302-s001.pdf]

RNA sequencing-based analysis of gallbladder cancer reveals the importance of the liver X receptor and lipid metabolism in gallbladder cancer

SUPPLEMENTARY TABLE

Supplementary Table S1: Total of 519 genes that were differentially expressed in GBC as compared with normal GB mucosa

See Supplementary File 1
